# Psychiatric trainees as second victims after exposure to patient suicide: a French qualitative study

**DOI:** 10.3389/fpsyt.2023.1308021

**Published:** 2023-12-18

**Authors:** Christine Durif-Bruckert, Bruno Cuvillier, Maxime Vieux, Benoit Chalancon, Philippe Villeval, Edouard Leaune

**Affiliations:** ^1^Université Lumière Lyon 2, Bron, France; ^2^Center for Suicide Prevention, Centre Hospitalier le Vinatier, Bron, France; ^3^Groupe d’Etude et de Prévention duSuicide, Saint-Benoit, France; ^4^RESHAPE, Inserm U1290, Université Claude Bernard Lyon 1, Lyon, France

**Keywords:** suicide, patient, psychiatry, mental health, medical education

## Abstract

**Background:**

The exposure to patient suicide (PS) has been identified as one of the most frequent and troubling professional experience for psychiatric trainees. Further studies are needed to better understand how residents cope with these experiences and the association between perceived support and the impacts of PS.

**Method:**

In this qualitative study, we aimed to assess the impact of exposure to PS during psychiatric residency on trainees’ professional career and practical experience. A total of 19 French psychiatric residents participated in 4 focus-groups performed between November 2017 and May 2019.

**Results:**

A total of 4 thematic clusters were identified through a five-step content analysis, namely: (a) reactions to the exposure; (b) coping strategies; (c) professional impact; and (d) prevention and postvention proposals. All participants described the critical impact of the support provided after PS, especially by their senior staff. Those who felt supported by their superior reported less negative impact, both in emotional and professional dimensions. Participants also shared proposals to improve the prevention and postvention issues related to the exposure to PS.

**Conclusion:**

We performed the first qualitative study based on focus groups on the impact of PS on psychiatric residents, which allowed for an in-depth understanding of the participants’ lived experiences of the exposure to PS. The narratives inform the need and means to implement prevention and postvention strategies designed to buffer the negative impact of the exposure to PS in psychiatric trainees.

## 1 Introduction

According to national surveys, 63 to 91.5% of psychiatrists have experienced patient suicide (PS), particularly in the early stages of their careers (1). Adverse medical events are recognized to be extremely challenging for healthcare practitioners (2). The concept of “second victim,” as introduced by Wu (3), describes emotional, cognitive, and behavioral reactions that healthcare professionals experience after adverse events. These reactions are associated with coping strategies that affect not only the second victims but also their colleagues and patients. Following such adverse events, second victims have reported both negative and constructive changes in their daily practice (2, 4). Experiencing adverse events during the initial stages of a career can prove to be exceedingly difficult, and lead to significant adjustments in the clinical practice of inexperienced professionals or trainees (5). Nevertheless, there is a paucity of literature about the effects of such events on early-career professionals, particularly those in medical training.

Psychiatric trainees commonly encounter PS, which has been identified as one of the most distressing experiences during their training (6, 7). Nearly half of all psychiatric trainees experience PS, especially during the first stage of their training, as reported in a recent literature review (8). The aftermath of PS may lead to high levels of trauma, emotional distress, and professional impacts (8). A significant portion of residents demonstrate clinical post-traumatic symptoms or encounter difficulties in managing their clinical practice for days and months after the event. This literature review reported a lack of institutional support, with almost a quarter of residents indicating no perceived support after PS in a Canadian study (9).

Interestingly, another group of substantial residents reported exposure to PS as a beneficial experience, mirroring the model of posttraumatic growth reported in people bereaved by suicide (10). However, little is known about the processes that help psychiatric residents cope with this adverse event. Further studies are needed to explore in depth the coping strategies used by residents following these experiences and the relationship between perceived support and the impact of PS.

In this qualitative study, we aimed to assess the impact of exposure to PS during psychiatric training on trainees’ professional careers and practice experiences. We aimed to assess how residents cope with PS in terms of emotional and professional impact, and their needs for institutional policies and support related to these experiences. A qualitative model embedded in a mixed methods study was considered an appropriate methodology for this purpose, as it allowed for an in-depth understanding of participants’ lived experiences of a complex phenomenon such as exposure to PS.

## 2 Method

### 2.1 Data collection

#### 2.1.1 The IMPACT-S study

IMPACT-S (*IMpact of PAtient suiCide during psychiatric Training – a mixed-method Study*) was a nationwide mixed-method study conducted in France from November 2017 to June 2019 and designed to assess the prevalence, emotional, traumatic, and professional impact of PS during psychiatry training by collecting qualitative and quantitative data from a diverse sample of psychiatry residents. According to the taxonomy outlined by Palinkas et al. (11), the structure of the IMPACT-S study utilized a sequential and complementary collection of quantitative and qualitative data. The study incorporated researchers from diverse fields, including psychiatry and mental health, social psychology, work psychology, health philosophy, and health anthropology. Their interest in the topic stemmed from their professional experience in psychiatric settings and expertise in healthcare and work organization research. The quantitative findings have been previously published (12).

#### 2.1.2 Focus-groups

For the qualitative part of the study, French psychiatry residents were sent an email via associative mailing lists, inviting them to participate in focus groups if they had been exposed to PS during their training. Focus groups are a qualitative research technique that effectively captures rich depictions of participants’ lived experiences and in-depth knowledge of complex phenomena (13). Focus groups also reflect researchers’ interest in social representations, as well as the views and positions of participants concerning a phenomenon while enabling an assessment of its social and institutional significance. Volunteers for participation contacted the principal investigator (EL) and were subsequently enrolled in the study. Funding was provided for the selected focus group venue, if deemed necessary. Focus groups were conducted in French and facilitated by two researchers specializing in social psychology and health anthropology (CDB) and social and work psychology (BC). Focus groups lasting between 1 h and one and a half hours were recorded and then transcribed. Results are presented in accordance with the *COnsolidated criteria for REporting Qualitative research* checklist (COREQ) (14).

### 2.2 Data analysis

According to previous literature (15, 16), a five-step content analysis was separately and independently performed by three authors (CDB, BC and MV). The five steps of the content analysis included (a) preparing the data (i.e., transcription of the interviews); (b) reading transcripts repeatedly to achieve immersion in the narratives and obtain a sense of the whole; (c) making notes on the transcript by listing the different types of information found in the text; (d) defining the unit of analysis using themes; and (e) developing a coding scheme to organize data in a comprehensible way. Content analysis is a frequently used method for studying complicated phenomena within qualitative research. The purpose of content analysis is to discover the underlying meanings of text by evaluating the significance of spoken or written language Content analysis has been described as “indigenous” to communication research because it examines data captured in messages and communications against observable events or individual characteristics (17).

According to the intramethod triangulation developed by Renz et al. (18), the fourth and fifth steps involved employing two distinct strategies, namely manual and computer-based techniques. Two authors (CDB and BC) employed a manual method, while the third author (MV) utilized NVivo software to conduct computer-based content analysis, which assisted in creating the coding scheme and defining the units of analysis. According to Renz et al. (18), a combination of manual and computer-based strategies as a method of data analysis triangulation can potentially generate more meaning from the data and enhance the inferences that researchers can make from the words and responses of participants. This approach has great potential for describing and inferring characteristics of verbal communication, especially in focus groups. Accordingly, the findings of the distinct analyses were then pooled together, discussed between four authors (CDB, BC, MV, and EL) and summarized. The focus groups were driven, transcribed, and analyzed in French. The verbatims were translated into English through an English language editing service for the article.

### 2.3 Enhancing rigor of the qualitative design

To enhance the rigor of our qualitative design, two main measures were employed, namely triangulation and saturation. Triangulation involves the utilization of numerous methods or data sources in qualitative research to develop a comprehensive understanding of the phenomena (19). Four types of triangulation have been identified by Denzin (19) and Patton (20): (a) method triangulation; (b) investigator triangulation; (c) theory triangulation; and (d) data source triangulation. The IMPACT-S study ensured all four types of triangulation. First, methodological and data triangulations were achieved through the mixed-method design employing a multidisciplinary approach that fosters active collaboration between researchers from diverse backgrounds. Second, the analysis process also underwent triangulation as three researchers (BC, CDB, and MV) independently performed the data analysis using two separate strategies. The results were then summarized and discussed with a third party (EL). Third, investigator and theoretical triangulations were also ensured by involving researchers from various disciplines, including social psychology (CDB, MV), work psychology (BC, PV), health anthropology (CDB), psychiatry (EL, BCh), and health philosophy (EL).

Saturation is a methodological approach aimed at ensuring the reliability and representativeness of data collected through qualitative research by ensuring that the collection of new data would not add new insights into the outcome of the study (21). The researchers confirmed that the final focus group did not introduce any additional themes. Based on the sample’s homogeneity (i.e., French psychiatric residents), we anticipated that 15 to 20 participants would be sufficient to reach data saturation. After conducting four focus groups with 19 participants, we decided to conclude the study as there were no new themes emerging in the final focus group.

### 2.4 Ethical concerns

The IMPACT-S study received ethical approval from the Ethics Committee of the Hospices Civils de Lyon and was funded by the Scientific Research Committee of the Vinatier Hospital Center and the Université Lumière Lyon 2 (funding number CSLV13).

## 3 Results

### 3.1 Characteristics of the participants

Table 1 “Characteristics of the participants” contains the age of the participants, their gender, current year of practice and the number of exposures to PS. A total of 19 French psychiatric residents participated in 4 focus groups performed between November 2017 and May 2019. Each focus group included between 3 and 7 participants. The mean age of the participants was 27.8 years. Twelve participants were women, and 8 were in their third year of residency. The vast majority have been exposed to inpatient suicides during their first year of residency.

**Table 1:** Characteristics of the participants.

Variable	*n*	Percentage
Age (mean)	19	27.8
Gender (female)	12	63.1
Current year of training		
First	3	15.8
Second	2	10.5
Third	8	42.1
Fourth	6	31.6
Year of training at exposure		
First	13	68.4
Second	5	26.3
Third	1	5.3
Fourth	0	0.0
Number of exposure(s)		
1	15	78.9
2 or more	4	21.0

### 3.2 Characteristics of the patients who died by suicide

Table 2 “Characteristics of the patients who died by suicide” contains the age of the patients, the place of their suicide and their status (inpatient or outpatient). Most of the patients involved in PS were inpatients, age between 35 and 65. PS mostly occurred in hospitals.

**Table 2:** Characteristics of the patients who died by suicide.

Variables	*n*	%
Age		
<18	1	5.3
18–35	4	21.1
36–65	11	57.9
<65	3	15.8
Inpatient		
Yes	17	89.5
No	2	10.5
Place of suicide		
Home	6	6.7
Hospital	8	42.1
Public place	4	21.1
NA	1	5.3

### 3.3 Content analysis

A total of 4 thematic clusters were identified through the duplicate data analysis, namely: (a) reactions to the exposure; (b) coping strategies; (c) professional impact; and (d) proposals for prevention and postvention. The verbatims of the participants for each theme are displayed in Table 3 (reactions to the exposure), Table 4 (coping strategies), Table 5 (professional impact) and Table 6 (proposals for prevention and postvention).

**Table 3:** Reactions to the exposure to patient suicide reported by the participants.

Subtheme	Verbatim quotes
Cognitive reactions	*“The recurring thought was to wonder: ‘What did I miss? What did I do wrong?’”*
	*“I found myself after two months of residency with a patient who had hanged herself and I was overwhelmed.”*
	*“Right away I said to myself: ‘Anyway, there was nothing else I could have done.’”*
	*“I made such irrational cause–effect links that made sense, sort of magical thoughts.”*
Emotional reactions	*“The feeling of guilt and shame was the most present.”* *“This made me feel more guilty as I asked myself: ‘Could I have done it differently?’”*
	*“I was very scared.”* *“It was a big surprise, a big shock the next morning.”*
	*“I felt very sad.”*
	*“I was particularly angry with the chief who brought the patient out.”*
Physical reactions	*“I was there wandering around the room. I did not know what to do.”*
	*“I was in a state of dissociation all night long.”*
	*“I rushed to the emergency room to get an oxygen bottle, thinking « there you go », feeling all the excitement of resuscitation.”*

**Table 4:** Coping strategies after patient suicide reported by the participants.

Subtheme	Category	Verbatim quotes
Social interactions	Support from the chiefs and senior psychiatrists	*“After my chief told me: ‘Well it’s tough, you have two suicides at once’, they all called me, one by one, the heads of other departments […]. I felt truly supported, helped on the situation.”* *“This is the time when everyone, all the heads of the other departments, send you a little text or a call, asking if you want to talk about it.”* *“And then my chiefs told me, ‘This is the unpredictable side, this is how it happened and there was nothing else we could do’.”*
	Support from colleagues	*“I could not discuss it with any senior. I spoke about it a little bit with this nurse, but in an informal way.”*
	Clinical staff meetings	*“The meeting truly helped me. Because it was another form of exchange not with an ‘alter ego’, in the sense of someone with whom I could truly identify, but people who knew the patient, who were also surprised and shocked by what happened the day before.”* *“We had a clinical staff meeting every Monday morning, with the whole team, so we turned that into a meeting to talk about this patient. I found it was great to have the senior doctor, the nurses and myself being there.”*
	Lack of support from the family and friends	*“The hardest part was my family’s reaction, as they are all doctors in my family. In addition, it was particularly tough when I told this to my mother who said: ‘But you know, in medicine there are a lot of patients who die’ and it is true.”* *“And then people from outside, relatives, family... They often understand but, finally, it is in fact difficult to truly comprehend when you are completely outside of health care.”* *“People do not truly understand the relationships that we can have with our patients.”*
	Meeting with the family of the deceased	*“I was scared, I did not want to meet this woman at all.”* *“I wasn’t truly reassuring to meet the family.”* *“And I think it was truly important, to be able to see the family, explain to them that we were sorry, that we were not expecting his suicide and that if we were expected we would have act differently. I truly think that having met the family, being able to express that we were sorry helped me a lot.”*
	Police inquiry	*“The police came later. It was quite an experience because frankly, with the way they act, you could only feel guilty.”* *“Yeah right, I felt assaulted by the police.”* *“And that truly, that thing about the police, it was as if we were judged right away, as if we were responsible for the suicide.”* *“The policemen were truly aggressive.”*
Self-regulation	Emotional and cognitive coping strategies	*“I found it very interesting to refocus on something medical and scientific.”* *“I told myself that it was necessary, then the word may be a little bit strong, “indifferent,”* i.e.*, detached from the effectiveness that can have one of my interviews.”* *“I felt it was my responsibility, and it was the first time a deceased patient had this effect on me since the beginning of my residency. […] I recall thinking at that time, ‘We missed something.’”* *“I bore all the responsibility: interviewing the family to explain the cause of death, the family suing the institution. I was entitled to all that. It was very complicated.”*
	Quest for meaning	*“However, at that moment, you tell yourself that you have a superpower in your hands, a superpower that you did not used at the right time. It may be true if I had seen him, I would have said “We will give him sedatives tonight.””* *“Maybe he had an anxious raptus in the morning, that is my theory”*

**Table 5:** Professional impact of patient suicide reported by the participants.

Subtheme	Verbatim quotes
Impact on their professional practice	*“It changed my way to provide care: I was controlling everything, all the time; I could no longer enter the rooms.”* *“Suicidal patients worry me so much now. At least when they proclaim it…it’s easier. We can expect it to happen.”* *“The fact that it happens during residency is a bad sign, I came to psychiatry so that there would be no suicides on earth.”* *“Now I realize that when a man with this profile comes into A&E, I’m extremely worried.”*
Impact on their training	*“You feel professionally inadequate, you tell yourself that you are not capable of doing this.”* *“The most disturbing about these events [patient suicides] is that we never know what it will do to us afterward.”* *“That discussion with another trainee helped me a lot, and even though I’m at the beginning of my residency, to adjust my definition of what this job is, to position myself in relation to that, and to absolutely want to continue […]. So, it made me choose the specialty of psychiatry again.”*
Change in their representation of the profession	*“We believe that psychiatry is a profession that saves lives, but it is also a confrontation to death.”* *“When this happens to you, it is neither fate nor fault, but bad luck, a professional hazard. It felt on me.”* *“It’s rare a job that is so strongly linked to death.”* *“We sometimes make mistakes, but no mistakes that have as much impact as a patient suicide.”*

**Table 6:** Proposals for prevention and postvention reported by the participants.

Subthemes	Verbatim quotes
Preparation to manage PS	*“How to manage a patient suicide is insufficiently taught during medical education, we are definitively not prepared to such an experience.”* *“Books are useful, but we also need someone who explains the experience to us, the lived experience: how to put a little distance, learn a little to ‘sail at sea’. It’s not only about books.”* *“We must prepare ourselves for our helplessness toward people who want to kill themselves.”*
Informal support from colleagues	*“What helped me was that we had a place for psychiatric residents. Every lunchtime I went to eat with my fellow residents. We were a small group and that changed everything.”* *“The debriefing with the team was important, because it meant having people to talk about it with and receiving the goodwill of the people around.”*
Formal support	*“I would have appreciated receiving a supervision, something more formal.”* *“It makes all the difference to have dedicated time, a regular and stable supervision.”*

PS, patient suicide.

#### 3.3.1 Reactions to the exposure

Table 3 “Reactions to the exposure to PS reported by the participants” contains verbatims for the three categories of reactions [i.e., (a) cognitive, (b) emotional, (c) and physical] reported by the participants. The participants reported experiencing emotional upheaval after exposure to PS, leaving them feeling overwhelmed and unable to cope. Guilt was the most reported emotion, with participants feeling personally and professionally responsible for the death of their patient.

Cognitive responses comprised cognitive processes that aid in comprehending an event. The objective to comprehend how and why the suicide occurred was a central point of these reactions, leading to repeated questions about the event and its causes.

*“The recurring thought was to wonder: ‘What did I miss? What did I do wrong?’”*

*“I found myself after two months of residency with a patient who had hanged herself and I was overwhelmed.”*

Trainees reported experiencing feelings of uselessness and confusion. Additionally, some trainees reported experiencing a post-traumatic dissociative state immediately after PS. Cognitive processes encompass both rational thoughts (i.e., medical, epidemiological, and/or scientific explanations) and irrational thoughts.

*“I made such irrational cause–effect links that made sense, sort of magical thoughts.”*

Encountering PS was described as an emotional upheaval, inducing a negative emotional state. Trainees frequently reported feeling guilt and shame, with strong feelings of having made mistakes. Additionally, there were reports of fear, shock, surprise, and sadness. Some trainees expressed feelings of anger, mainly directed toward their superior or institution.

*“The feeling of guilt and shame was the most present.”*

*“I was particularly angry with the chief who brought the patient out.”*

Physical reactions after PS included difficulty in taking action immediately. Trainees reported feeling lost and inactive, experiencing sleep troubles, hyperarousal, and physical dissociation. Resuscitation techniques may cause excitement in the immediate aftermath of PS.

*“I was in a state of dissociation all night long”*

*“I was there wandering around the room. I didn’t know what to do.”*

#### 3.3.2 Coping strategies

Table 4 “Coping strategies after patient suicide reported by the participants” contains verbatims for the two following types of coping strategies reported by our sample after PS: (a) social interactions and (b) self-regulation.

Regarding social interactions following these events, the importance of support from direct senior staff was emphasized. A comprehensive and reassuring approach by the senior staff was helpful for the affected trainees in two ways: managing expected professional activities after the event and providing a friendly environment for emotional debriefing. When trainees were not receiving adequate support from their superiors, they reported significant levels of guilt, feelings of being at fault, and psychological distress.

*“After my chief told me: ‘Well it’s tough, you have two suicides at once’, they all called me, one by one, the heads of other departments […]. I felt truly supported, helped on the situation.”*

*“And then my chiefs told me, ‘This is the unpredictable side, this is how it happened and there was nothing else we could do’.”*

Peer support was found to play a crucial role in the process of coping with PS. Informal discussions or formal meetings with colleagues provided emotional debriefing, which was described as beneficial. The absence of peer support may reinforce feelings of isolation and guilt.

*“I couldn’t discuss it with any senior. I spoke about it a little bit with this nurse, but in an informal way.”*

*“We had a clinical staff meeting every Monday morning, with the whole team, so we turned that into a meeting to talk about this patient. I found it was great to have the senior doctor, the nurses and myself being there.”*

A lack of support from their family and friends was reported as the trainees struggled to explain the event and its impact to their relatives and to receive adequate support from them.

*“And then people from outside, relatives, family... They often understand but, finally, it is in fact difficult to truly comprehend when you are completely outside of health care.”*

Two events were reported as particularly challenging for residents who were exposed to PS. The first was contacting the family of the deceased patient; although it provided relief to residents, it also caused anxiety.

*“I was scared, I did not want to meet this woman at all.”*

*“And I think it was truly important, to be able to see the family, explain to them that we were sorry, that we were not expecting his suicide and that if we were expected we would have act differently. I truly think that having met the family, being able to express that we were sorry helped me a lot.”*

The second was to handle administrative or police investigations, which could intensify emotions of guilt and blame. The police investigation can be a traumatic experience, amplifying the sense of having made mistakes.

*“The police came later. It was quite an experience because frankly, with the way they act, you could only feel guilty.”*

*“The policemen were truly aggressive.”*

Regarding self-regulation, the most frequent strategies reported were negative and include self-blame, self-depreciation, rationalization, detachment, or externalization. Usually, the quest to understand the feelings caused by the event led to a search for the cause of the suicide. Three main types of self-regulation were described by trainees: self-attribution (i.e., medical error, self-blame), external-attribution to the institution (i.e., lack of resources, organizational difficulties, team error), and external-attribution to the diagnosis and the patient. Trainees report that managing their sense of responsibility in the case of a presumed “medical error” was particularly difficult.

*“I found it very interesting to refocus on something medical and scientific.”*

*“I bore all the responsibility: interviewing the family to explain the cause of death, the family suing the institution. I was entitled to all that. It was very complicated.”*

#### 3.3.3 Professional impact

Table 5 “Professional impact of patient suicide reported by the participants” contains verbatims for the three following categories of professional impact: (a) modifications in their professional practice, (b) an impact on their training, and (c) a change in their social representation of the profession.

In the immediate period after exposure to PS, psychiatric trainees reported significant changes in their practice, including better suicidal risk evaluations, longer consultations, and increased duration of hospitalizations. Long-term changes may be occurring in their practice for some of them, particularly in the management of patients with suicidal ideation. Difficulty with managing relationships with suicidal patients was also described as a change in professional attitudes. Several participants reported that exposure to PS was a “bad sign” for their future careers and that they had difficulty managing the uncertainty in their practice.

*“It changed my way to provide care: I was controlling everything, all the time; I could no longer enter the rooms.”*

*“Now I realize that when a man with this profile comes into A&E, I'm extremely worried.”*

Regarding their training, PS was described as a professional trial that raises questions about residents’ professional commitment and competencies. Exposure to PS may provoke introspective questions about their own competencies and career path. Nonetheless, most residents reported that exposure to PS strengthened their determination and enthusiasm toward psychiatry and individuals with mental disorders.

*“You feel professionally inadequate, you tell yourself that you are not capable of doing this.”*

*“That discussion with another trainee helped me a lot, and even though I'm at the beginning of my residency, to adjust my definition of what this job is, to position myself in relation to that, and to absolutely want to continue […]. So, it made me choose the specialty of psychiatry again.”*

Concerning social representations, trainees reported that encountering PS may lead to a professional crisis, impacting their representations of psychiatric practice. Negative representations of the psychiatric institution include blaming the institution and the loss of meaning induced by organizational functioning. In particular, the professional ideal may be diminished.

*“We believe that psychiatry is a profession that saves lives, but it is also a confrontation to death.”*

*“When this happens to you, it is neither fate nor fault, but bad luck, a professional hazard. It felt on me.”*

#### 3.3.4 Proposals for prevention and postvention

Table 6 “Proposals for prevention and postvention reported by the participants” contains verbatims for the three following proposals reported by our sample: (a) preparation to manage PS, (b) informal support from colleagues, and (c) formal support.

Residents expressed the necessity for educational programs dedicated to exposure to PS. They emphasized the requirement for practical, hands-on learning, as opposed to purely theoretical instruction, focusing specifically on PS exposure. These programs should encompass a comprehensive understanding of the practical and emotional dimensions of PS management for both themselves and their teams.

*“How to manage a patient suicide is insufficiently taught during medical education, we are definitively not prepared to such an experience.”*

*“We must prepare ourselves for our helplessness toward people who want to kill themselves.”*

The participants reported that engaging in informal conversations with their peers was very beneficial to them, as it allowed them to express themselves freely about their exposure to PS and the emotional impact they experienced. Most of the participants also expressed feelings of isolation after their exposure to PS, highlighting the need for a friendly environment in which they could express and share their experiences. Participation in focus groups enabled them to reduce their sense of stigma and responsibility.

*“What helped me was that we had a place for psychiatric residents. Every lunchtime I went to eat with my fellow residents. We were a small group and that changed everything.”*

*“The debriefing with the team was important, because it meant having people to talk about it with and receiving the goodwill of the people around.”*

Residents expressed a demand for sufficient support after experiencing PS. This support should consider both personal and professional issues related to the exposure. They specifically emphasized the necessity for protocols and institutional assistance from superiors and teachers. Additionally, they suggested the provision of free psychotherapeutic counseling or supervision.

*“I would have appreciated receiving a supervision, something more formal.”*

*“It makes all the difference to have dedicated time, a regular and stable supervision.”*

## 4 Discussion

We conducted the first qualitative study that details the effect of PS on psychiatric trainees. Prior research on this topic relied on quantitative designs, surveys, or single case reports of trainees’ experiences (8). Our utilization of a qualitative approach facilitated the gathering of in-depth empirical data on the effect of PS within this vulnerable population. Our study aligns with previous research on the effects of the exposure to PS on psychiatric trainees and presents new findings that may inform prevention and postvention interventions aimed at preparing and supporting trainees during times of PS exposure. We provide insights into the significant emotional and professional impact of PS exposure on psychiatric residents. The trainees primarily reported employing negative self-regulation strategies, including self-blame and self-depreciation, that were linked to feelings of guilt and anxiety. Specifically, the trainees experienced guilt as they felt accountable for the patient’s death, leading to sensations of professional failure. Moreover, all participants highlighted the crucial significance of the support provided after PS, particularly from their senior staff. Those who perceived a lack of support from their senior staff reported experiencing adverse effects in both their emotional and professional spheres. Additionally, participants suggested potential solutions to address prevention and postvention concerns regarding exposure to PS during psychiatric training.

Our findings align with prior research on the emotional, traumatic, and professional implications experienced by psychiatric residents following PS (8, 12, 22). Indeed, earlier studies reported that psychiatric residents display elevated rates of posttraumatic reactions and emotional distress after encountering PS as reported in a recent systematic review (8). Furthermore, some investigations in Canada and Switzerland have determined that these reactions are more intense in residents in comparison to senior psychiatrists (9, 23). The professional impact of PS is noteworthy (8), as reported by our sample. It was found that their professional ideal was lessened as a result. For instance, participants reported that they entered psychiatric training with the goal of suicide prevention and decreasing suicide rates so that, after exposure to PS, they expressed feelings of personal and professional disappointment and professional incompetence. Furthermore, the stage of training has a critical impact, as younger trainees are known to exhibit more negative effects on their practice (1, 8), including a higher propensity to abandon their training. Consistent with our quantitative results (12), with found no variation in the impact based on the method of suicide used by the patients.

The trainees reported the lack of preparedness they felt toward encountering PS. Only a limited number of studies have previously assessed the effectiveness of educational programs dedicated to mitigating the impact of PS on trainees in the United States (24–26). A proposition of the participants in our study was oriented toward prevention programs during their training. According to our findings, we can identify various skills that need to be addressed in prevention programs: (1) educating psychiatric trainees about the widespread prevalence of exposure to PS during psychiatric training; (2) educating trainees about the traumatic, emotional, and professional consequences they may experience after PS; (3) teaching adequate skills to prevent suicidal behavior, especially in inpatient settings; (4) teaching adequate skills to cope with the aftermath of exposure, i.e., provide accessible resources to support trainees in managing the impact of the exposure to PS. These resources should focus on (a) providing knowledge on existing supports available after PS; (b) mitigating the effects of stigmatization on affected trainees; (c) managing communication with the families of the deceased; and (d) teaching skills to manage emotional, traumatic, and professional impacts after exposure (such as seeking formal or informal support, accepting emotional distress, and recognizing negative mental health outcomes like acute stress disorder).

Several trainees in our sample reported a lack of support after PS. However, other participants shared interesting experiences regarding the support provided after PS that can aid in building postvention programs. Notably, residents found participation in a focus group on the topic of PS to be a beneficial experience. They reported a desire to share their own feelings and distress with peers in a welcoming atmosphere. However, focus groups were not intended for structured debriefing. As a result, postvention programs dedicated to psychiatric residents could incorporate peer-support sessions. During these sessions, residents may share their experiences through structured debriefing (24). Employing a team-based debriefing, involving both the trainee and the entire team, can be a useful strategy in mitigating negative emotional and professional outcomes for trainees (27). According to a recent study assessing a postvention protocol among adult psychiatry trainees in the United States (28), support from attendings who had previously experienced an adverse event and from the program director was reported to be more helpful than other types of support. In addition, a resource has been produced in the United Kingdom by the *Royal College of Psychiatrists* (29) to aid psychiatrists in their recovery from PS. The resource recommends meeting with a senior clinician who comprehends the impact of PS and can provide confidential advice and support, as well as joining a confidential reflective practice group or space to process the effects of PS. In the study conducted by Agrawal et al. in the United States (28), residents also reported that debriefing with their supervisors was beneficial after PS.

Compared to other professions, psychiatric trainees emphasized the crucial involvement of senior staff in their management of PS. The feeling of being supported by a senior staff was found to be instrumental in implementing coping and emotional regulation strategies following PS. As demonstrated by previous studies in Canada and the United Kingdom (9, 30), many psychiatric trainees have reported a significant absence of support from their superiors, leading to feelings of anger as these trainees expected their senior staff and institutions to offer them necessary emotional and practical support. In Switzerland, Wurst et al. (31) reported that psychiatric trainees were significantly more likely to report negative professional outcomes following PS which can be worsened by a lack of support from senior staff. In the same time, supporting a trainee affected by PS can be a challenging task for senior psychiatrists, as they may not be adequately prepared to handle PS themselves and may experience similar psychological or professional impacts as the trainee. Several potential solutions could be proposed, including implementing systematic training for all senior psychiatrists who supervise psychiatric trainees to better manage PS, or providing formal support from the chief-resident for both psychiatric trainees and their supervising senior psychiatrists. A comprehensive training program for senior psychiatrists could include the following components: (1) educating senior psychiatrists on the high prevalence of exposure to PS during psychiatric training; (2) instructing psychiatrists on the emotional, professional, and traumatic effects experienced by trainees following PS; and (3) providing psychiatrists with the necessary skills to support psychiatric trainees after the exposure (e.g., knowledge of available resources, supporting exposed trainees experiencing stigma, managing relationships with the families of deceased individuals, offering formal and informal support).

The first strength of our study is the innovative methodology used to assess the lived experience of psychiatric residents when encountering PS. We conducted the first qualitative study conducted on this topic and provided additional pertinent evidence regarding the impact of PS on psychiatric trainees and the measures required to prevent negative outcomes after the exposure.

However, our study has several limitations. First, the qualitative design employing focus groups in this study may limit the representativity of our results as we included a small number of participants. Nevertheless, our findings are consistent with those of prior research, and the focus groups were included in a mixed-method investigation where quantitative results from a large sample were previously published (12). Furthermore, the use of triangulation and saturation enhanced the credibility of our results. Second, the use of focus groups can introduce biases in data collection as they may give priority to the loudest voices. Nevertheless, the researchers paid particular attention in addressing this concern. Specifically, we made sure that all participants in each focus group were able to express themselves and recount their personal experiences. Moreover, our sample was marked by a high degree of homogeneity and consensus within the group that can be attributed to their shared professional identity and age, as well as exposure to the same event during their training as young professionals. To gain a better insight into the impact of PS on psychiatric trainees, further research should consider exploring the exposure to PS in psychiatric training through individual in-depth interviews. Third, it is important to note that only French psychiatric trainees were included in this study. The generalizability of our results should therefore be viewed with caution regarding the idiosyncrasies of psychiatric residency in France in comparison to other countries. Finally, it is possible that our sample was affected by selection bias. The residents who participated in the study may have been more comfortable discussing their experiences of PS during the focus group, which could have introduced biases into their narratives.

## 5 Conclusion

We conducted a qualitative study to investigate the effects of PS on psychiatric residents. Interestingly, our study narratives provide a more detailed insight into the impact of PS and underscore the need for prevention and postvention programs aiming to mitigate the negative impact on psychiatric trainees in the aftermath of PS. Postvention strategies might encompass peer support sessions with other affected trainees, formal meetings with senior staff, and team-based debriefings that involve the entire team. The structured debriefing sessions during team-based meetings could serve as a platform for residents to share their experiences.

## Data availability statement

The raw data supporting the conclusions of this article will be made available by the authors, without undue reservation.

## Ethics statement

The studies involving humans were approved by the Ethics Committee of the Hospices Civils de Lyon. The studies were conducted in accordance with the local legislation and institutional requirements. The participants provided their written informed consent to participate in this study.

## Author contributions

CD-B: Conceptualization, Data curation, Formal analysis, Funding acquisition, Investigation, Methodology, Resources, Validation, Visualization, Writing – original draft. BrC: Conceptualization, Data curation, Formal analysis, Funding acquisition, Investigation, Methodology, Validation, Writing – original draft. MV: Data curation, Formal analysis, Investigation, Writing – review & editing. BeC: Investigation, Validation, Visualization, Writing – review & editing. PV: Validation, Writing – review & editing. EL: Conceptualization, Data curation, Formal analysis, Funding acquisition, Investigation, Methodology, Resources, Supervision, Validation, Visualization, Writing – original draft.
